# A systematic review of responsible stewardship of research and health data from Indigenous communities

**DOI:** 10.1038/s41746-025-01902-w

**Published:** 2025-08-05

**Authors:** Alec J. Calac, Timothy K. Mackey

**Affiliations:** 1 UC San Diego School of Medicine, San Diego, CA USA; 2 Global Health Policy and Data Institute, San Diego, CA USA; 3 UC San Diego Department of Anthropology, San Diego, CA USA

**Keywords:** Medical research, Scientific community, Social sciences

## Abstract

There is growing recognition of the moral and legal authority of Indigenous Peoples to regulate research and other matters that involve their communities under the principle of Indigenous Data Sovereignty (IDS). This systematic review has two aims: (1) detail IDS considerations and practices in health research in the United States and other global contexts; and (2) identify frameworks that operationalize IDS practices for responsible conduct of research and use of data-driven technologies. Our review returned 41 relevant articles detailing specific considerations for the collection, access, and use of Indigenous data, and sub-themes such as cultural and regional considerations. More than half of the articles articulated a theoretical framework or detailed set of guidelines for using Indigenous data, with two especially focused on digital data considerations. Results indicate that intentional engagement with Indigenous researchers and communities will minimize harm and maximize benefits for all participating in research and technology development.

## Introduction

Indigenous Data Sovereignty (IDS), defined as the authority of an Indigenous entity to govern the collection, ownership, and application of their data, can enhance traditional community-based participatory research (CBPR) approaches by directly engaging Indigenous communities in all stages of the research process^1–9^. IDS aims to protect Indigenous communities from the harms of research misconduct and ensure that research is conducted appropriately from the perspective of both research review boards and community members^4,7^. In the United States, an understanding of IDS is crucial to engaging with American Indian or Alaska Native (AI/AN) governments because they are considered sovereign entities with the legal authority to regulate research, public health practice, and the operation of data systems on their lands^5,6^. The application of IDS to research and public health practice is also important in other global Indigenous contexts as our data-driven world increasingly relies on digital tools and systems to improve human health^9^.

When public health agencies disregard IDS and impede the timely provision of public health data to AI/AN communities, it can have serious public health implications^10,11^. In the United States, state and federal agencies refused to share COVID-19 case and population-level data with Tribal Epidemiology Centers and Tribal governments (both of which are federally-designated public health authorities), which limited their ability to conduct timely contact tracing and identify COVID-19 exposures in non-residential settings^12^. Even when AI/AN data are available, non-tribal data systems are known to misclassify AI/AN individuals as “non-Hispanic White” or “Other,” which leads to disease underreporting and the diversion of resources available to AI/AN communities experiencing public health crises^13–19^.

Existing research studies have examined social and structural drivers of health disparities among AI/AN communities, including those that concern data governance^7,20,21^. A number of frameworks have also been proposed that seek to operationalize IDS in the conduct of medical and public research, but have limited their scope to protections concerning physical biospecimens (e.g., blood), genomics, and the conduct of CBPR on tribal lands^22–24^. The applicability of IDS in digital health is poorly understood and more-so rooted in theoretical application^25^. Therefore, it is important to understand the present strengths and limitations of IDS in research and public health practice and identify strategies to engage IDS in the development and use of emerging digital technologies in medicine and research.

The aims and intentions of this systematic review are to detail IDS considerations and practices in CBPR and related health-focused research in the United States and other global contexts (e.g., First Nations, Māori) and identify frameworks that operationalize IDS practices for responsible conduct of research and use of data-driven technologies with Indigenous communities^26^.

## Results

**Fig. 1:**
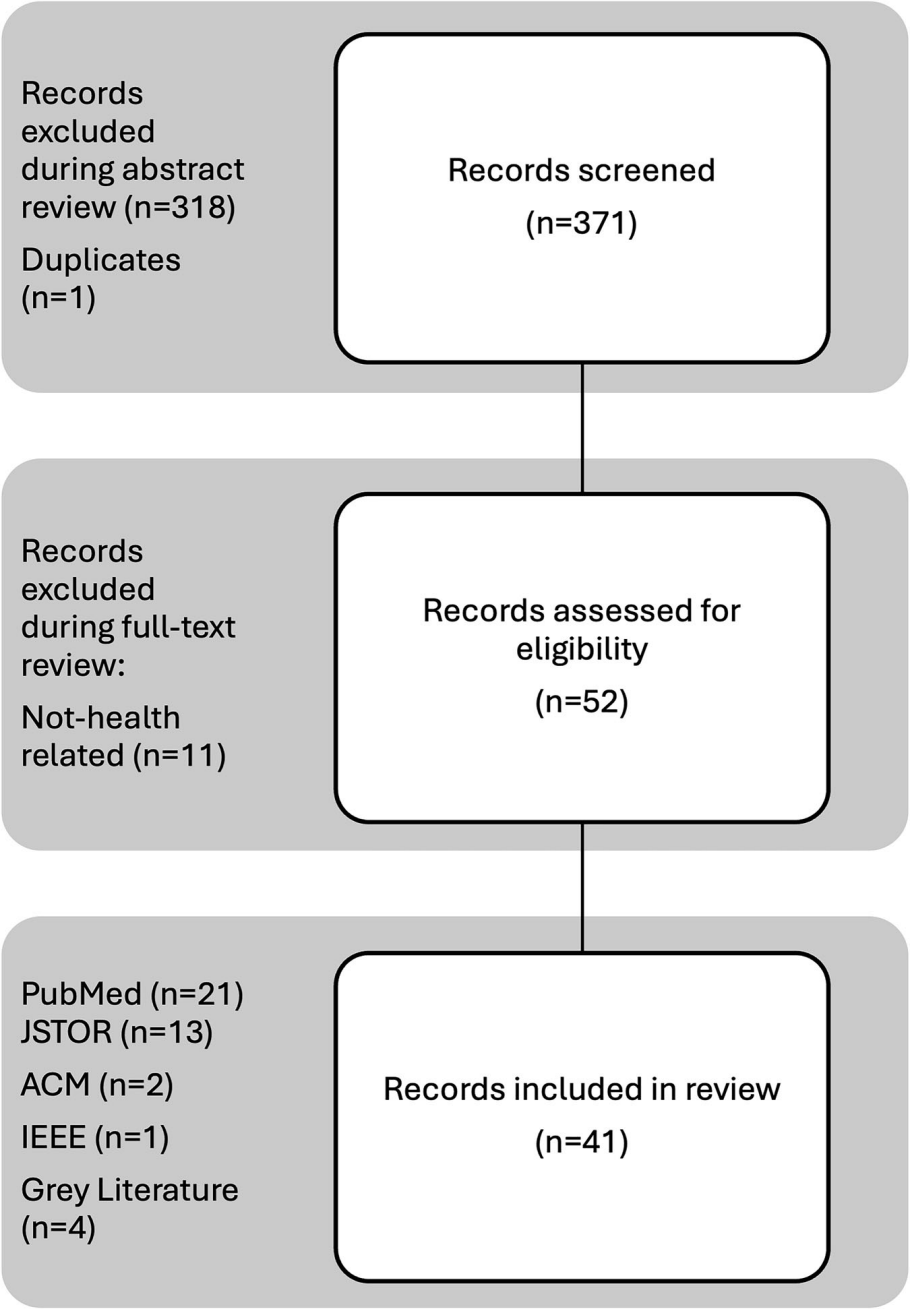
PRISMA flowchart, detailing article review process. This systematic review adhered to PRISMA guidelines as applicable for the article content types included in the review. A total of 371 articles published between 2013 and 2024 were identified and screened on PubMed, IEEE Explore, ACM, and JSTOR. After abstract screening, which involved removal of duplicates and confirmation of article type, a total of 53 full-text articles were reviewed based on this study’s systematic review inclusion and exclusion criteria. After further review of the full text, 41 indexed, peer-reviewed articles were deemed eligible for analysis and synthesis of results (11 excluded for non-health focus), 21 from PubMed, 13 from JSTOR, 2 from ACM, and 1 from IEEE Explore^27–38^. Additionally, 4 articles consisting of policy briefs and communiqués from the gray literature were reviewed and came from The University of Arizona Native Nations Institute (U.S.), First Nations Information Governance Center (Canada), and Te Mana Raraunga Māori Data Sovereignty Network (New Zealand), and are discussed in the eSupplement. Article types primarily consisted of original research articles (*n* = 20, 49%), followed by essays, commentaries, and perspectives (*n* = 12, 29%), reviews (*n* = 5, 12%), and policy briefs (*n* = 4, 10%).

A total of 371 articles published between 2013 and 2024 were identified and screened on PubMed, Institute of Electrical and Electronics Engineers (IEEE) Explore, Association for Computing Machinery (ACM), and JSTOR. Articles influenced by the COVID-19 pandemic were published between March 2020 and December 2024. After abstract screening, which involved removal of duplicates and confirmation of article type, a total of 53 full-text articles were reviewed based on this study’s systematic review inclusion and exclusion criteria. After further review of the full text, 41 indexed, peer-reviewed articles were deemed eligible for analysis and synthesis of results (11 excluded for non-health focus), 21 from PubMed, 13 from JSTOR, 2 from ACM, and 1 from IEEE Explore (shown in Fig. 1)^27–38^. Additionally, 4 articles consisting of policy briefs and communiqués from the gray literature were reviewed and came from The University of Arizona Native Nations Institute (U.S.), First Nations Information Governance Center (Canada), and Te Mana Raraunga Māori Data Sovereignty Network (New Zealand), and are discussed in the eSupplement. Article types primarily consisted of original research articles (*n* = 20, 49%), followed by essays, commentaries, and perspectives (*n* = 12, 29%), reviews (*n* = 5, 12%), and policy briefs (*n* = 4, 10%). Study designs, key findings, and other review-relevant summary information are presented in Table 1.

**Table 1:** Systematic review published between 2012 and 2024, detailing article type, study design, regional context, use of a framework or set of best practices, technology-related applications of IDS, and key findings

Article	Type	Study design	Region	Guidelines	Technology or health area	Key findings
Abadie and Heaney (2015)	Research	Semi-structured interviews (*n* = 16) with non-reservation-based AI/AN individuals.	U.S.	–	Biobanking	• Concerns over genetic discrimination based on race. • Debate over the merits of collective consent over individual consent.
Bardill (2014)	Review	–	–	–	Genomics	• Ancient DNA is one of many facets of contemporary Native American communities.
Bolnick et al. (2016)	Review	–	–	–	Genomics	• New technologies reduce the need for destructive analyses of ancestral remains to obtain DNA.
Champagne (2015)	Article	–	–	–	–	• Tribal communities want research to benefit their lives. Possible for Indigenous communities to convene IRBs when recognized as legal, self-governing entities.
Duarte et al. (2019)	Review	Qualitative and quantitative analyses in accord with a feminist ethics of care.	–	–	–	• Data has many facets or dimensions. • Indigenous science favors holism over reductivism.
Garrison et al. (2019)	Research	Semi-structured interviews (*n* = 14) with health professionals, policy experts, and tribal leaders.	U.S.	–	Genomics	• Concerns about open vs. closed data access structures • Identified need for tribally-operated data repositories, rather than federal ones.
Hiratsuka et al. (2020)	Research	Organizational regional partner profiles (*n* = 4).	U.S.	Center for the Ethics of Indigenous Genomic Research	Genomics	• Varying tribal organizational capacities to conduct and manage external research.
Kuhn et al. (2020)	Research	Content analysis of active and deactivated tribal IRBs (*n* = 6).	U.S.	X	–	• Tribal IRB requirements may differ from academic IRB applications. • Each tribe maintains unique research data management requirements.
Minthorn et al. (2019)	Research	Indigenous researcher perspectives (*n* = 4).	U.S.	X	–	• Signing memoranda of understanding can foster reciprocal research relationships. • Tribal sovereignty creates political autonomy from the settler-state.
Pearson et al. (2014)	Research	RCT assessing research training module effectiveness (*n* = 40) with reservation-based AI/AN individuals.	U.S.	X	–	• Research training in AI/AN contexts can promote cultural safety and minimize risks to individuals and communities.
Radin (2017)	Article	–	U.S.	–	Machine Learning	• Digital data will outlive the individuals who generated it. How can data systems be held accountable to living persons? • Machine learning is a neutral tool that is biased by the data and people who use it. Digital ethics guidelines must be developed.
Radin (2018)	Review	–	–	–	Genomics	• Tribal “refusal” to participate in research studies can lead to dialogs about how to improve study design.
TallBear (2013)	Article	–	–	–	Genomics	• Indigenous identity has cultural, genetic, political, and social aspects. • DNA testing has no direct bearing on tribal membership.
Caron et al. (2023)	Research	Key informant interviews (*n* = 32) and focus groups (*n* = 7) with First Nations leaders, health directors, health practitioners, and community members.	CAN	OCAP Principles	Genomics	• Consent mechanisms should respect First Nations governance. • Capacity building and sustainability should be addressed in research proposals. • Communication and cultural safety can engender trust with community members.
Hudson et al. (2023)	Article	–	–	OCAP Principles, CARE Principles	–	• Indigenous communities have the right to know who has access to their data and define how it is used, among other rights.
Baldwin et al. (2023)	Research	Interviews (*n* = 92) and talking circles (*n* = 13) with AI/AN first responders, educators, elders, and substance use recovery researchers.	–	CARE Principles	–	• Indigenous determinants of health and sovereignty can affect community resilience, mental health, and well-being. • Data governance frameworks facilitate external research partnerships.
Kowal et al. (2022)	Article	–	–	UNDRIP	Genomics	• Community partnerships should guide ancient DNA research conduct. • Open data conflicts with Indigenous Data Sovereignty.
Carroll, Plevel et al. (2022)	Research	Review of Tribal research legislation, policy, and administrative materials (*n* = 26 tribes) using a legal epidemiology approach.	–	CARE Principles	Genomics	• Use of Indigenous governance frameworks can facilitate benefit sharing and research reciprocity. • Tribes primarily regulate research through legislation and policy, with emphasis on restricting commodification of research data.
White et al. (2022)	Review	–	–	X	–	• Inappropriate interpretation of AI/AN datasets can promote stereotypes and bias. • Large-scale datasets do not preclude the need to adapt study design to safeguard AI/AN communities.
Carroll, Suina et al. (2022)	Research	Consensus panel (*n* = 8) of Indigenous scholars.	–	World Health Organization Social Determinants of Health Framework	–	• Western determinants of health conflict with Indigenous determinants of health and well-being. • Research frameworks and policy instruments must always account for local context.
Carroll, Garba et al. (2022)	Article	–	NA	CARE Principles, FAIR Principles	Genetics	• Global Indigenous Data Alliance is operationalizing CARE within repositories, ethics frameworks, and UN open-data guidance documents.
Haring et al. (2021)	Article	–	–	X	Genomics	• Public health crises do not minimize the need to respect Indigenous Data Sovereignty. • Data-use agreements provide a legal basis for tribes to seek recourse for research misconduct.
Huyser et al. (2021)	Research	State-level analysis of AI/AN COVID-19 mortality rate reporting.	–	–	Public Health Surveillance	• 26 of 50 U.S. states reported AI/AN COVID-19 mortality rates. • Exclusion of AI/AN data figures hinders tribal advocacy efforts.
Gartner et al. (2021)	Article	–	–	X	–	• AI/AN data aggregation limits the local applicability of research studies. • AI/AN data aggregation can mask regional health disparities.
Haozous et al. (2021)	Article	–	–	X	–	• Belmont Report may be inadequate for the conduct of research with urban AI/AN communities. • Individual AI/AN participation in research can have tribal implications.
Credo and Ingram (2021)	Research	Case study on three tribal research partnerships.	–	–	–	• Identifies actions that promote productive tribal research collaborations. • Data ownership and dissemination must be proactively addressed at the pre-research application stage.
Small-Rodriguez and Akee (2021)	Research	State-level analysis of reporting categories for AI/AN decedents.	U.S.	X	Data Linkage	• Racial misclassification is prevalent among AI/AN decedents when compared across different data systems. • No current standard for the collection of AI/AN tribal membership data in vital statistics systems.
Rowe et al. (2021)	Research	Retrospective analysis of conference session proceedings.	–	SEEDS Principles, CARE Principles	Data Linkage	• Indigenous population health data linkages increase data quality. • SEEDS Principles are intended to complement CARE Principles.
Carroll, Akee et al. (2021)	Article	–	–	CARE Principles, OCAP Principles	–	• Data, including tribal identifiers, should be validated for responsible decision-making. • Community-controlled data infrastructures promote Indigenous Data Sovereignty.
Yellow Horse and Huyser (2021)	Research	N/A	–	–	–	• Increasing data availability and quality can help tribes address public health challenges. • Disaggregated tribal data should be suppressed from external parties.
James et al. (2014)	Research	Retrospective analysis of conference session proceedings.	U.S.	–	–	• Agencies, such as the NIH, can encourage grantees to accept tribal oversight of research conduct. • Tribal laws and cultural interests should be given priority during study design negotiations with tribes.
Harding et al. (2012)	Article	–	U.S.	X	–	• There are numerous codes and ethics, and data-sharing agreement models for tribal and First Nations research. • Presents a model Indigenous material data-sharing agreement.
Mackey et al. (2022)	Research	–	U.S.	X	Blockchain	• Modularity of blockchain-based infrastructure provides for customization to local AI/AN contexts. • Decentralized data governance mechanisms increase local oversight and data management.
Doğan and Wood (2023)	Research	Case study and interviews (*n* = 3) with Indigenous environmental practitioners.	U.S.	CARE Principles	GIS	• Indigenous communities eagerly support collective or material benefits for participating in research or sharing data. Management of environmental data is just as important as human research and health data.
Zong and Matias (2024)	Research	Design of the conceptual framework.	U.S.	Data Refusal from Below, OCAP Principles	–	• Collective autonomy accounts for multiple individuals and communities. Refusal can be used as a basis for descriptive, evaluative, and generative power.
Bowen and Hinze (2022)	Research	Retrospective application of the conceptual framework.	NZ	Te Mana o te Raraunga	Biosensors	• Indigenous Data Sovereignty is reliant on people, not just the design of data systems.

"X" denotes presence of general principles, not otherwise specified as a named framework or list.

Nearly half (*n* = 20) of included articles were original research articles that explored health-related applications of IDS, ranging from public health surveillance to data governance to responsible conduct of CBPR, primarily in North American Indigenous contexts (U.S. and Canada)^39,40^. One article presented the findings of a randomized controlled trial assessing the acceptability and relevance of research training modules adapted for an AI/AN-focused research project among AI/AN community members, and several (*n* = 8, 20%) performed critical analyses of tribal institutional review board (T-IRB) and AI/AN-focused research review protocols or conducted interviews with regional T-IRB members, community members, and individuals interfacing with AI/AN communities^24,41–48^. Regional perspectives from AI/AN individuals, Tribal leaders, academics, and professionals broadly covered the Pacific Northwest, Midwest, and Southwestern regions of the United States, and also made specific recommendations premised on recognition of an AI/AN Tribe or Villages as governmental entities^39–41,43,44,49–51^.

Challenges to tribal public health authority and the exercise of IDS during the course of the COVID-19 pandemic were frequently referenced (*n* = 10, 24%), likely due to increased discussions on which entities should regulate and oversee the collection, management, and access of public health data obtained from Indigenous Peoples^11,12,40,46,51–56^. A number of articles focused on the ethical, legal, and social considerations of collecting genomic and genetic data from Indigenous Peoples (*n* = 11, 27%), and were less focused on considerations for any other types of data generated by or collected from AI/AN communities^23,24,39,43,47,50,57–61^. Several articles compared and contrasted a data system framework that incorporated IDS principles onto another framework, namely the FAIR and CARE frameworks (defined later in this review)^8,11,62,63^. Two articles detailed specific guidelines or principles for safeguarding digital health and online multimedia data generated by Tribal members living away from Tribal lands^26,50^. Additional sections characterize IDS principles and frameworks, research considerations, and technology use cases.

### IDS Principles

The articles discussed in this review primarily focus on the proposed collection, access, and use of research and health data from Indigenous communities. Other relevant sub-themes focused on how the misuse of data from Indigenous communities can perpetuate health disparities and conflict with Indigenous cultures and origin narratives.

More than half of the included articles (*n* = 23) articulated a theoretical framework or detailed a set of cultural and practice guidelines for using Indigenous data. Several (*n* = 5) with direct relevance to the management of digital data from AI/AN communities are presented in Table 2^25,62–64^. These purposefully operationalize IDS in different global contexts.

**Table 2:** Selected research frameworks incorporating Indigenous Data Sovereignty

Framework	Principles	Literature
CARE Principles for Indigenous Data Governance	(**C**)ollective Benefit, (**A**)uthority to Control, (**R**)esponsibility, (**E**)thics	Carroll et al.^25^
SEEDS Principles	(**S**)elf-determination, (**E**)xercise Sovereignty, (**E**)thics, (**D**)ata Stewardship and Governance, (**S**)upport Reconciliation	Rowe et al.^62^
OCAP First Nations Principles	(**O**)wnership, (**C**)ontrol, (**A**)ccess, (**P**)ossession	Scnarch^63^
Data Refusal from Below	**Autonomy**: Individual and Collective, **Time**: Reactive or Proactive, **Power**: Foreclosing Possibility or Creating Possibility, **Cost**: Accepting Cost or Reducing/Redistributing Cost	Zong and Matias^64^
Te Mana o te Raraunga	**Assessment of Data Use**: **Whakapapa** (level of relationship), **Pukenga** (level of expertise), **Kaitiaki** (level of authority), **Wananga** (level of responsibility).**Assessment of Data Users**: **Tika** (level of value), **Pono** (level of trust), **Wairua** (nature of application), **Mauri** (level of originality).	Bowen and Hinze^66^

The CARE Principles for Indigenous Data Governance, designed and validated by a group of AI/AN and Indigenous researchers, are meant to encourage the responsible use of big data and associated technologies^25^. Key considerations under the first CARE principle (**C**ollective Benefit) include focuses on inclusive technology development and innovation, improved data governance and user engagement, and equitable outcomes^25,48^. Frequent engagement with and iterative feedback from end-users of data-driven technologies can result in updates and adjustments that maximize benefit and return on investment of Indigenous data^65,66^. Key considerations under the second CARE principle (**A**uthority to Control) focus on the recognition of Indigenous rights and interests, collecting data that furthers Indigenous self-governance, and prioritizing Indigenous governance of data, especially when data are housed at external academic centers and biorepositories^25,67^. Considerations under the third CARE principle (**R**esponsibility) focus on positive relationships, promoting employment opportunities and research capacity, and respect for Indigenous languages and worldviews^25,41,62,67^. The fourth CARE principle (**E**thics) includes a focus on minimizing harm and maximizing benefit, justice, and restrictions on secondary use of data^25,44,48^.

The SEEDS Principles are focused on population health data linkages and complement the CARE framework’s sociopolitical implications of contrasting “governance of data” (a set of practices) with “data for governance,” which focuses on data-driven policymaking^11,40,44,62^. The first two principles (Self-Determination and Exercise Sovereignty) acknowledge the fundamental rights of AI/AN and Indigenous communities to oversee the conduct of ethical Indigenous health research (third principle), implement robust data governance mechanisms (fourth principle), and production of meaningful research findings that support reconciliation (fifth principle) with non-Indigenous societies^11,62^. By linking together Indigenous and non-Indigenous data systems, a fuller picture of Indigenous health outcomes can be obtained^17,62^. This information can then be used by policymakers and Indigenous rights advocates to secure resources and financial support.

The OCAP Principles were developed by the First Nations Information Governance Center in Canada and are specific to North American Indigenous contexts^63^. The principles of Ownership, Control, Access, and Possession have been implemented into policies and procedures concerning the release of provincial data to First Nations communities and researchers^63^. This prior implementation of the OCAP principles was paramount to a robust response to the COVID-19 pandemic among First Nations communities and medical personnel^11^.

The framework “Data Refusal from Below” explores the practice of refusal in a North American Indigenous context through considerations of autonomy, time, power, and cost^64^. Community refusal is distinct from non-consent and functions to increase research utility and relevance to Indigenous knowledge in contemporary society^57^. Refusal is also a form of resistance against policies and research practices that do not honor the authority and data sovereignty of Indigenous communities^47,50^. In the case of digital technology systems, researchers and Indigenous leaders may want to consider how the constructs in these frameworks map to specific data structures, protocols, and algorithms for the purposes of IDS and responsible data management^50^.

The final framework is presented through a data and computer science perspective and the New Zealand Indigenous context. In Bowen and Hinze^66^ the Te Mana o te Raraguna Framework is mapped onto the implementation of the Hakituri project, which incorporates data from occupational work biosensors, environmental hazard monitoring systems, and feedback systems routed to supervisory parties at worksites^66^. This Framework details measurable concepts and characteristics that can be qualitatively and quantitatively assessed as being met at the levels of low, medium, and high by researchers and community members^66^. Data users are assessed for their level of relationship, expertise, authority, and responsibility specific to the aims of the implementation, and data use cases are assessed for their level of value, trust, originality, and nature of application^66^. This Māori framework promotes IDS by facilitating participatory data design wherein user-identified needs and challenges form the basis for the development of technology-driven solutions, rather than trying to use a one-size-fits-all design irrespective of local contexts^66^.

Together, all of these frameworks provide examples of IDS being operationalized in AI/AN and global Indigenous contexts and the potential benefits that can be derived from external partnerships.

### Considerations for Use of Indigenous data

Researchers believe that data originating from or generated by tribal communities, especially genomic data, can provide unique insights into the evolution of humanity^57,58,60^. However, many in the scientific community believe that such data has no value until researchers spend significant time and resources to transform the data into something that is considered “valuable” by researchers^56^. Given that there are many types of AI/AN data that may interest researchers, ranging from the anthropological to biological, it is important to specify the tribal governmental entities and community-based groups that can regulate or co-manage the collection of AI/AN data by external entities and outside researchers on and off tribal lands and scope of IDS (see Table 3)^9,42,47,50^.

**Table 3:** Key applicability issues associated with the collection of AI/AN data

Description	Applicability	Other considerations
Entities that regulate research involving tribal members on tribal lands (not necessarily away from tribal lands)	Tribal councils (governing bodies of AI/AN Tribes and Villages), research review boards (IRBs and T-IRBs), and U.S. governmental agencies, such as the Indian Health Service (IHS)^42,43,51^. Additionally, Federal laws governing the protection of human research participants (45 CFR § 46–) apply to AI/AN-based research regulatory bodies, including requirements to maintain active registration in state and federal systems^26,45^.	Notably, tribal governments can exercise their right to self-governance and enact rules and laws that meet and exceed these specified protections in an effort not just to protect research participants, but also the entire community where the research is occurring^4,67^. The need for these robust protections was precipitated by numerous instances of questionable research practices involving AI/AN individuals and communities^39,47,57,58^.
Informed consent (IC) is especially important when conducting research with AI/AN communities^58^.	Prior to engaging with potential research participants, researchers should have well-defined research questions that focus primarily on identified needs in the community, and sign data management plans with AI/AN research authorities, limiting secondary use of research data from human subjects, supplementing any agreements also signed between T-IRBs and researchers’ affiliate institution(s)^8,43,50,57^.	Informed consent approaches must include respect for cultural protocols, such as considering which figures are able to authorize the disclosure and sharing of data sought by researchers, such as elders and tribal leaders^46^. IC must also respect the privacy of AI/AN knowledge systems and the importance of protocols for the handling of sensitive knowledge, especially when making decisions that can have lasting generational implications^39,45^.
Scope of data collection	The scope of data collection from prospective AI/AN research participants living or accessing services on tribal lands is determined not just by a T-IRB or IHS IRB, but also by consulting tribal leaders and health program officers before a formal IRB application^24,59,67^. Tribal authorities can exercise their “right to refuse” to release data from the community, which is an internationally-recognized practice affirmed by the United Nations Declaration on the Rights of Indigenous Peoples (UNDRIP) and the principle of free, prior, and informed consent that recognizes the AI/AN right to self-determination^8,62,86^. Importantly, this right can be exercised at any stage of the research process (collection to dissemination of findings), not just by a participant but also by tribal authorities^57,64^.	The approvals provided and agreements made with these bodies limit the outflow or extraction of potentially sensitive data from an AI/AN community^42^. For example, this provides an opportunity for tribal health authorities to consider whether specific demographic variables (e.g., tribal affiliation) can be collected and reported, or if a more general racial identifier will be used, such as AI/AN or Native American^43,86^.

#### Data access, ownership, knowledge systems, and relationality

Notably, data management challenges often arise when non-AI/AN entities (e.g., local and state governments) collect data from AI/AN communities, especially during times of public health crisis, and refuse to share that data with tribal public health authorities^52^. Tribal governments and tribal epidemiology centers have the same public health authority as local and state governments, as designated by Congress, but these groups do not readily share primary source data with tribal authorities^68^. In the midst of COVID-19, AI/AN morbidity and mortality data were misclassified as other, multi-racial, or not reported at all due to the low proportion of AI/AN COVID-19 cases relative to other racial and ethnic groups^52,53^. It was estimated that just more than half of U.S. states (*n* = 26) were able to report COVID-19 mortality rates for AI/AN communities, despite there being 574 federally-recognized tribes in 37 states and disparate COVID-19 mortality and morbidity rates compared to the non-Hispanic White population^53^.

Other considerations include collecting AI/AN research data electronically and also via paper forms, an important consideration given long-standing challenges in accessing reliable Internet coverage among AI/AN communities and limited sharing of data between local, state, tribal, and federal data systems^41,46,53^. However, attention should be given to the accuracy of paper-based responses transcribed into electronic research databases because of the risk of human error. While paper-based data collection may seem outdated, it allows AI/AN research participants to limit the collection of metadata that can be used for other purposes not related to primary data collection and analysis, and promotes greater participation of rural and urban-underserved AI/AN communities in human subjects research^50–59^.

Data collected from an AI/AN community may be stored at an academic institution, managing entity (e.g., biorepository at a non-profit institution), or by tribal health authorities^43,45^. Regardless, it is important that all data maintain relationality with the Indigenous context they came from, as researchers may seek to deconstruct or parse data for the purposes of analysis, but such a reductivist approach may conflict with Indigenous knowledge systems^59,62^. Indigenous researchers have proposed alternative approaches, such as “weaving together” Indigenous and Western methods of inquiry and knowledge generation to strengthen research findings and make them relevant to AI/AN communities^56^. Notably, even data analyses and interpretations are subject to AI/AN data management protections because there is potential for misuse of data and biospecimens^62^. In human genomics and genetics research, investigators have been criticized by AI/AN scientists and communities for using “destructive” analytical tools (e.g., chemical solvents, drilling) that physically damage ancestral remains and objects of cultural significance^58,69^. Data digitalization can minimize the need for destructive analyses, so long as data management plans are in place that ensure ownership after the conclusion of research^50^.

Tribal interests and priorities largely shape permitted research involving AI/AN individuals, communities, and entities^44,45^. Mismanagement of AI/AN research data has led to AI/AN governments barring specific types of research (e.g., 2002 Navajo Nation moratorium on genetic research studies) and prioritizing research that is tied to real issues faced by AI/AN communities, rather than research for the sake of research^39,47,58^. This system maximizes the potential benefit of the research for AI/AN communities and promotes cultural safety by prohibiting research that can challenge the validity of AI/AN cultural knowledge^58,60,61^. Of note, these considerations vary from community to community, with some allowing for investigation into their genetic origins, while others may only permit genetics research to address present-day health disparities^58^.

Indigenous researchers have importantly characterized restrictions on permitted research and non-participation in external (non-AI/AN-based) research parties as a matter of *refusal*^57,64^. This concept challenges external researchers to better engage with AI/AN communities and identify research questions that are tied to identified community needs, rather than institutional priorities or requirements of a funding agency^57^. The affirmative exercise of refusal at all stages of the research process also has relevance in an increasingly digital world, wherein the iterative or step-wise design of machine learning algorithms and artificial intelligence can benefit from operationalizing IDS and purposefully incorporating community feedback^44,50,59,64^.

#### Responsible data governance, metadata, and regulatory authority

Tribal governments have used numerous mechanisms to protect and exert ownership over their health and research data, including the application of intellectual and cultural property claims and use of relevant state and federal laws, such as the Native American Graves Protection and Repatriation Act in the United States, when improper research practices have been identified by AI/AN communities^43,57,59–61^. With IDS, the focus is not solely on individual ownership of data, but also community ownership of data, because a single AI/AN or Indigenous individual does not necessarily have the authority to represent the interests and priorities of their community^62^. This principle is most applicable to tribal members living on tribal lands because T-IRBs have authority over all research conducted on reservations^26^. However, these legal protections do not prevent or discourage tribal members from participating in research conducted off tribal lands^26^. However, tribal governance and exercise of IDS over academic and related research conducted outside of the boundaries of tribal lands is limited and may not be respected by the parties overseeing the research, which can perpetuate AI/AN health disparities^26,47^.

There are outstanding questions of data ownership pertaining to non-recognized AI/AN governmental entities, Native Hawaiian or Pacific Islander governments (e.g., entities with no federal recognition or a process to do so), or those in the active process of seeking recognition in the US^39,43^. While these groups are unable to use federal statutes and regulations spacific to federally-recognized AI/AN governmental entities, the United Nations Declaration on the Rights of Indigenous Peoples (UNDRIP) articulates inherent rights for Indigenous entities that should ultimately be respected by signatories of this UN declaration, such as the US^67^. The UNDRIP may also help non-recognized AI/AN governments and urban AI/AN communities secure similar research protections as given to federally-recognized AI/AN governments, even if these guidelines and principles are not enshrined in state and federal research statutes and regulations^48^.

When data are available from AI/AN communities, unique identifiers are often removed (e.g., tribal affiliation, health facilities) to protect community^59,62^. Other practices in furtherance of IDS goals may include removing discussion of sensitive cultural practices and spiritual knowledge incidentally collected on audio recordings, limiting access to primary data to specified team members, and specifying policies and procedures for the return of data and dissemination of findings to the community for policymaking and health promotion to protect Indigenous knowledge from being exploited^42,55^. These considerations are typically part of a larger data management plan that is agreed upon by all parties at the outset of the proposed research and can be modified by a T-IRB^8,42^. Use of data should also be limited to individuals who have completed training on responsible conduct of research and any other applicable tribal requirements, including cultural sensitivity training and certificates of confidentiality regarding AI/AN cultural practices^42,44,56^. Training modules from the Collaborative IRB Training Initiative are frequently completed by persons involved in biomedical, behavioral, and social sciences research, but these modules are not readily adapted for research studies involving AI/AN and other Indigenous communities^41^.

Tribal data-sharing and data-use agreements are typically signed between academic institutions, researchers, T-IRBs, and, in some cases, the Indian Health Service^45^. These agreements specify how research findings are to be disseminated (e.g., conferences, publication) and often require internal review of manuscripts and abstracts before submission^42,43,56^. Federal law and tribal law provide authority to T-IRBs and governmental agencies to request manuscript language changes, non-disclosure of sensitive data analyses, and removal of information that could identify an AI/AN community engaged in research^54,56^. Other actions include limiting access to primary datasets, return of all data, culturally-appropriate destruction of biospecimens (e.g., hair, blood), and presentation of research findings to the community to facilitate knowledge exchange and capacity-building^22,42,54,57^.

#### Open-access challenges, competing interests, and tribal leader engagement

Federally-funded research studies may require data submission to open-environment biorepositories that lack the robust protections and governance structures used by AI/AN research authorities^39,43,54^. The pooling of data from numerous AI/AN communities has the risk of generalizing research findings from one community to another or an unrelated community on the basis of race alone, rather than on genetics and other relevant factors^43,46,51^. Further, the federally-appointed membership of the committees that oversee data requests from these repositories usually does not have expertise in AI/AN-focused data management^43^. This creates tension between non-AI/AN-engaged researchers who favor “open” research environments and AI/AN communities who favor “closed” research environments or “use-by-consent” to safeguard against misuse of data that may perpetuate health disparities and stereotyping of AI/AN lifestyles and practices through a Western lens^47,67,69^.

Engaging Tribal leaders throughout the entire research process, especially in subsequent policy and program development, remains a challenge. The implementation of the U.S. National Institutes of Health All of Us Research Program was informed by a Tribal Advisory Committee (TAC) that was hastily formed after grantees began regional recruitment in areas with large AI/AN populations without first consulting Tribal governments^24^. Policies resulting from the work of the TAC included: (1) a pause on active recruitment from AI/AN populations until consultations could be conducted nationwide; (2) no specification of a participants’ AI/AN tribal affiliation unless the tribal government has an agreement with All of Us; and (3) barring nationwide access to AI/AN participant data until clear mechanisms for AI/AN data governance could be developed^70^. Some tribal leaders responded to the All of Us Research Program by supporting the creation of the Native BioData Consortium on the lands of the Cheyenne River Sioux Nation. The Consortium is the first Indigenous-led U.S. biorepository working to ensure that emerging technologies and large-scale health research studies, like the All of Us Research Program, benefit all AI/AN communities^64^.

The primary purpose of IDS is to use lessons learned from past misuses of research data to protect communities from exploitation and safeguard resources^64^. Open data environments do not readily use mechanisms that protect the rights and interests of Tribal governments, which has encouraged organizations like the IEEE to identify practices for the provenance and responsible use of digital Indigenous data^8^. Some argue that open data has a dual benefit of reducing the need to create new data and the need to conduct destructive analyses, but AI/AN leaders and communities have concerns about the longevity of these data^50^. These discussions warrant considering sociocultural reasons for data use and whether these needs outweigh the inherent rights of AI/AN communities to limit access to their data^69^.

The movement for open data and inherent longevity of clinical, genetic, and digital data raise questions about informed consent and the “politics of reuse” across the entire research timeline, especially in Indigenous communities^50^. Tribes can permit varying types of consent, such as consent specific to a single research study or broad consent, which is specific to data reuse across multiple research studies, either with or without requirements for additional T-IRB review and participant consent^43,50^. Broad consent conflicts with IDS because it minimizes the involvement of T-IRB, and it may increase the risk of harm and loss of data to third parties with interests to co-opt and commercially exploit AI/AN health data^54^. In the U.S., these concerns were raised during the peak of the COVID-19 pandemic when AI/AN communities participated in novel vaccine development and relied on third-party COVID-19 antigen testing services without time to review data use agreements^40,54^.

### Technology use cases incorporating IDS

Increasing recognition of the importance of IDS has led to the design of technologies and data systems that respect its principles and the real-world application of IDS. Joffrion and Fernández^38^ present a case study with the Penobscot Nation in the U.S. state of Maine, highlighting how the CARE Framework was used to collect, store, and disseminate data from geographic information system (GIS) forestry analyses, monitoring environmental health, and vegetation density^71^. Bowen and Hinze^66^ detail IDS design considerations and data flows for the Māori Hakituri Project, which collects streaming personal sensor data from forestry workers (e.g., physical activity) and contextual data from the work environment (e.g., environmental sensors) on a closed-network system^66^. These data are then processed and routed back to workers and managers in the form of real-time feedback, such as hazard alerts and fatigue warnings^66^. In this case, research data management plans and data use agreements were used to operationalize IDS and delineate varying levels of data access^66^. Notably, there were no discussions about the use of these data for internal research and development by the manufacturers of these technologies as specified in the relevant Terms and Conditions of Service and Privacy policies, which may warrant future investigation and action by tribal authorities.

Before the COVID-19 pandemic, many proposed applications of IDS focused on genetics and genomics research, including biobanking operations and concerns regarding digital data longevity and informed consent. In an effort to bolster tribal precision medicine infrastructure, the Native BioData Consortium (as described in Mackey et al.^72^) is working to incorporate privacy preserving data governance protocols into the operation of their genomic biorepository, after concerns were raised with regard to federal handling of AI/AN genetic data by the U.S. National Institutes of Health and Mayo Clinic^24,72^. A Community Advisory Board comprised of individuals from the U.S. Northern Plains Region provides input on data access requests, potential benefits sharing, and handling of biospecimens^72,73^. Other IDS discussions have explored the potential benefits and harms of training machine learning algorithms on data attributable to AI/AN communities in open-data environments^50^. Radin^50^ highlights how the Pima Indians Diabetes Dataset has helped researchers and AI/AN communities model and identify obesity and diabetes trends among tribal members in the Southwestern U.S., then highlights a concerning use of the same dataset by non-AI/AN data scientists to train algorithms in New York City to predict the likelihood of catastrophic sewer explosions and manhole fires^50^. This is an example of how AI/AN data can be transformed and used in a way that the original context is lost, and no benefit derived from use of the data can return to the community^59,62^.

The literature identified key considerations for the responsible collection, management, and use of research, health, and digital data generated by Indigenous Peoples. It appears that new technologies and systems, such as machine learning algorithms and artificial intelligence, are being rapidly designed without explicit rules and regulations that operationalize IDS and protect Indigenous Peoples from exploitation and cultural harm in the era of big data. Emerging digital health technologies have clear potential to benefit communities, but only when they are used in a manner that respects the communities that provide their foundational data.

## Discussion

This review identified 41 records, including peer-reviewed articles (*n* = 37) and policy briefs and communiqués in the gray literature (*n* = 4, see eSupplement) published over the past 10 years, focused on key considerations for the responsible conduct of research in AI/AN and global Indigenous contexts^74–77^. Most articles discussing IDS were identified in the PubMed and JSTOR libraries. The gray literature and ACM and IEEE libraries were useful in identifying articles, frameworks, and policy briefs discussing barriers and proposed solutions to facilitate IDS in global contexts. Most articles also touched on cultural and governmental considerations for the handling of data, with some proposing theoretical frameworks and constructs rooted in Indigenous knowledge systems and the responsible stewardship of data pertaining to Indigenous and AI/AN persons, communities, and cultures.

Previous reviews examining IDS have primarily focused on ancient DNA, AI/AN population genomics, and the complex intersection of AI/AN race and biology^57,58,61^. This review expands IDS applications to also consider biobanking, machine learning and artificial intelligence, public health surveillance, data systems linkages, blockchain, biosensors, and GISs. Though outside the time period this systematic review was conducted (our review search was conducted in March 2024), a late 2024 perspective article by Cordes et al. also highlighted the tensions associated with open health data and IDS movements led by Indigenous peoples in the context of digital health^20^. The article highlights specific challenges of Indigenous data governance in the context of data collection, use, and management, similar to discussed in this review, while also advocating for concrete actions to improve availability, access, accuracy, beneficial data use, sociocultural relevance, collaboration, and building capacity and infrastructure aligned with IDS and practice within Indigenous communities^20^.

Crucially, the push for equitable health outcomes acknowledges a long history of research exploitation and data extraction from Indigenous communities^54,59^. A related focus in Indigenous health research that parallels data sovereignty, especially for national projects involving Māori communities, is the concept of equal explanatory power, wherein research outcomes equally benefit Indigenous and non-Indigenous communities due to equal sampling, rather than recruitment based on population size^78^. To realize Indigenous health equity, data must always be collected, reported, and tied to social and political movements to improve disparities, not to reinforce and worsen them^64,79^. In that way, data is more than an asset or resource. Instead, it acts as a tool for sovereignty, empowerment, and cultural revitalization^59^. This purposeful use of physical and digital data is applicable not just to Indigenous communities but may also be adapted for use in other non-Indigenous contexts where power dynamics exist between the majority and minority^80^.

While some literature focused on protections for digital data, it was clear that principles of IDS can be applied to responsible stewardship of all types of data affiliated with or owned by an Indigenous community, ranging from the cultural to the biological^50,59,64^. These use cases were well described, as virtually all of the literature was written by authors with an Indigenous affiliation or research groups working closely with Indigenous communities. Further, despite an extensive search of the published literature, no collected articles criticized the movement for IDS nor presented counter-arguments against community ownership and governance over research data. Continuing to operationalize IDS in practice, whether required or not by a legal entity or regulatory authority, will ultimately lead to better research outcomes and benefits for all parties involved.

Future investigations of IDS should monitor how novel technologies, such as machine learning and artificial intelligence, and legally-binding accords, such as data management plans, can be used to promote IDS and responsible data governance. These issues were of particular concern for tribal leaders when data-use agreements were signed between Indigenous communities and companies advertising COVID-19 diagnostic services and therapeutics research with no restrictions on secondary use of biospecimens^54^. Another topic of interest, especially to tribal public health practitioners, is mitigating health disparities among Indigenous youth and adolescents. For example, the tobacco industry likely uses AI/AN cultural messaging and iconography to promote tobacco sales and foster tobacco-related disparities among AI/AN youth and adolescents^81^. In the era of big data, researchers respecting IDS may consider surveillance of tobacco-related content on social media using machine learning and other data-driven methodologies to tailor interventions for a population that increasingly uses social media platforms^50,82–86^. These data can help Tribes respond to incidents of public health significance without the need to make the general, non-Indigenous population aware of health disparities faced by AI/AN youth and adolescents.

It appears that new digital health technologies, such as machine learning algorithms and artificial intelligence, are being rapidly designed without input or guidance from the communities that will interface with these systems for personal use, research, health-related, or commercial purposes. It will be important to develop rules and regulations that operationalize IDS and protect Indigenous Peoples from exploitation and cultural harm in the era of big data. While some argue that these systems will be designed to protect consumer rights and privacy, there are community-level safety and cultural concerns regarding the risk for data loss and misuse of sensitive knowledge. Ideating and co-creating on the potential development and application of novel digital health technologies and systems between Indigenous researchers, leaders, and non-Indigenous data scientists and engineers will help prioritize the interests and priorities of Indigenous Peoples and maximize the potential benefit that these technologies can bring to these communities. Attention will also need to be dedicated to narrowing the digital divide and ensuring that communities have access and appropriate training to benefit from these emerging technologies.

## Methods

This systematic review collates multi-level perspectives on IDS, especially in clinical and research-based settings^26^. Additional topics include cultural considerations for the handling of sensitive data and relevant frameworks that support the responsible use of Indigenous health data. This systematic review was not prospectively registered in a systematic review registry but was informed by consultations with a community advisory board as part of a project examining the use of digital technologies to manage Indigenous genomic data. To mitigate the potential for bias, we strictly adhered to the Preferred Reporting Items for Systematic Reviews and Meta-Analyses (PRISMA) reporting guidelines as applicable to our review. Author AJC independently screened and read full-text articles, presented findings to Author TKM, and a final consensus for inclusion was reached between Authors AJC and TKM. All PRISMA steps, ranging from the literature search strategy, article consensus, relevant study characteristics, study group and results synthesis, and certainty of evidence, are detailed in Fig. 1 and the eSupplement.

We conducted an interdisciplinary review across multiple databases in March 2024 (Supplementary Table 1). We considered original research articles, reviews, editorials, and viewpoints in four indexed databases: PubMed (Medline), ACM Digital Library, Journal Storage Digital Library (JSTOR), and IEEE Explore Digital Library. We selected these databases for the broadest coverage of the interdisciplinary nature of the research topic, including, but not limited to, the umbrella of health sciences, law, engineering, social sciences, and technology. Conference abstracts were excluded because of the variability in peer review and inclusion in databases.

Database searches were performed using combinations of the Medical Subject Headings (MeSH) “American Indian or Alaska Native” (ID: D044467) and the non-MeSH terms “IDS” and “Health”. Keywords were entered into the Title/Abstract field advanced search settings for PubMed, ACM, IEEE Explore, and JSTOR. The University of Arizona Native Nations Institute (U.S.), First Nations Information Governance Center (Canada), and Te Mana Raraunga Māori Data Sovereignty Network (New Zealand) were also queried for gray literature responsive to the query “Indigenous Data Sovereignty” and IDS, as these organizations are recognized both nationally and globally for leading research, policy, and advocacy discussions around IDS. A 10-year review period was considered appropriate for our focus on research frameworks that have emerged as a result of recent public health jurisdictional challenges concerned with IDS.

We sought to include research articles, essays, commentaries, perspectives, and related non-original research articles, acknowledging that AI/AN and global Indigenous perspectives are widely interdisciplinary and diverse across the medical, engineering, and social sciences. Key findings and outcomes were identified by conducting an abstract review and review of results and implications sections, if needed. This review was primarily focused on the specific ethical, legal, and social considerations and applicability of IDS in AI/AN research and public health infrastructure in the United States, also assessing IDS considerations for the design and operation of digital health technologies. Articles responsive to our search methodology specific to other global Indigenous contexts (e.g., Canada, New Zealand) were also included in our content synthesis if they met these inclusion criteria. We excluded screened articles that did not detail specific guidelines or considerations for applying IDS to the collection, management, and use of health data originating from Indigenous communities. Author AJC independently screened and read full-text articles, presented findings to Author TKM, and a final consensus for inclusion was reached between Authors AJC and TKM. Key findings were identified in the abstract sections of the identified articles and reported in Table 1.

## Supplementary information

Supplementary Information

## Data Availability

The datasets generated and/or analyzed during the current study are not publicly available because the dataset consists of extracted data from published studies and includes notes that are a part of ongoing analyses, but are available from the corresponding author upon reasonable request.

## Code availability

During the data extraction phase, Microsoft excel (version 16.98, Mac edition) was used to document references, remove duplicates, screen references for title and abstract, and list articles for inclusion in the review. No other custom code, scripts, or other tools were used in the generation of analysis of data for this study.
